# Supplementary material for: Group identification drives brain integration for collective performance
Source: eLife. 2025 Jun 24;13:RP100000. doi: 10.7554/eLife.100000 (PMC12187129; doi:10.7554/eLife.100000)
Supplement: Supplementary file 1. [file elife-100000-supp1.docx]

**Table S1.** MNI coordinate Position of 3×5 optode probe set

|  | **MNI coordinate Position** | | |  |  |
| --- | --- | --- | --- | --- | --- |
|  | **x** | **y** | **z** | BrodmanArea (Chris rorden' MRIcro) | **Percentage** |
| **CH01** | 36 | 40 | 42 | 9 - Dorsolateral prefrontal cortex | 0.967742 |
| **CH02** | 13 | 50 | 46 | 9 - Dorsolateral prefrontal cortex | 1 |
| **CH03** | -11 | 50 | 45 | 9 - Dorsolateral prefrontal cortex | 1 |
| **CH04** | -35 | 40 | 42 | 9 - Dorsolateral prefrontal cortex | 0.913043 |
| **CH05** | 47 | 42 | 28 | 45 - pars triangularis Broca's area | 0.94382 |
| **CH06** | 26 | 57 | 33 | 46 - Dorsolateral prefrontal cortex | 0.504854 |
| **CH07** | 2 | 59 | 34 | 10 - Frontopolar area | 0.934211 |
| **CH08** | -23 | 56 | 33 | 6 - Dorsolateral prefrontal cortex | 0.654762 |
| **CH09** | -45 | 42 | 27 | 45 - pars triangularis Broca's area | 0.92233 |
| **CH10** | 38 | 19 | 58 | 46 - Dorsolateral prefrontal cortex | 0.789474 |
| **CH11** | 14 | 68 | 23 | 10 - Frontopolar area | 1 |
| **CH12** | -13 | 67 | 22 | 10 - Frontopolar area | 1 |
| **CH13** | -35 | 58 | 19 | 46 - Dorsolateral prefrontal cortex | 0.916667 |
| **CH14** | 47 | 53 | 2 | 46 - Dorsolateral prefrontal cortex | 1 |
| **CH15** | 27 | 68 | 8 | 10 - Frontopolar area | 0.934211 |
| **CH16** | 2 | 68 | 9 | 10 - Frontopolar area | 1 |
| **CH17** | -24 | 68 | 8 | 10 - Frontopolar area | 0.972973 |
| **CH18** | -45 | 53 | 1 | 46 - Dorsolateral prefrontal cortex | 1 |
| **CH19** | 38 | 63 | -7 | 10 - Frontopolar area | 0.622222 |
| **CH20** | 15 | 71 | -3 | 11 - Orbitofrontal area | 0.615385 |
| **CH21** | -13 | 71 | -3 | 11 - Orbitofrontal area | 0.770833 |
| **CH22** | -36 | 63 | -7 | 10 - Frontopolar area | 0.586207 |
