# Supplementary material for: Group identification drives brain integration for collective performance
Source: eLife. 2025 Jun 24;13:RP100000. doi: 10.7554/eLife.100000 (PMC12187129; doi:10.7554/eLife.100000)
Supplement: Supplementary file 2. [file elife-100000-supp2.docx]

**Table S2.** MNI coordinate Position of 2×​4 optode probe set

|  | **MNI coordinate Position** | | |  |  |
| --- | --- | --- | --- | --- | --- |
|  | **x** | **y** | **z** | **BrodmanArea (Chris rorden' MRIcro)** | **Percentage** |
| **CH01** | -34 | 2 | 66 | 2 - Primary Somatosensory Cortex | 0.65079 |
| **CH02** | -45 | -26 | 67 | 22 - Superior Temporal Gyrus | 0.48867 |
| **CH03** | -46 | -54 | 59 | 39 - Angular gyrus, part of Wernicke's area | 0.67845 |
| **CH04** | -41 | 14 | 59 | 43 - Subcentral area | 0.83019 |
| **CH05** | -51 | -14 | 58 | 22 - Superior Temporal Gyrus | 0.75851 |
| **CH06** | -57 | -43 | 54 | 37 - Fusiform gyrus | 0.44554 |
| **CH07** | -57 | 43 | 54 | 19 - V3 | 0.8 |
| **CH08** | -54 | 2 | 50 | 21 - Middle Temporal gyrus | 0.54808 |
| **CH09** | -64 | -27 | 45 | 21 - Middle Temporal gyrus | 0.47187 |
| **CH10** | -63 | -51 | 37 | 37 - Fusiform gyrus | 1 |
