# Supplementary material for: Group identification drives brain integration for collective performance
Source: eLife. 2025 Jun 24;13:RP100000. doi: 10.7554/eLife.100000 (PMC12187129; doi:10.7554/eLife.100000)
Supplement: Supplementary file 3. [file elife-100000-supp3.docx]

**The results of HbR.**

We attempted to verify if the pattern of associated results was comparable to that of HbO when the analyses of HbR were conducted. First, by performing one-sample *t*-tests for GNS, we observed a significantly increased GNS in the OFC (CH20, *t* = 2.11, *p* = 0.030, FDR corrected; CH21, *t* = 6.76, *p* < 0.001, FDR corrected). We then conducted independent *t*-test on GNS in OFC (CH21), indicating a significant difference between the High and Low Group Identification groups (*t*_58_ = 2.04, *p* = 0.040, FDR corrected).
